# Platelet Storage Quality, Plasticizer Migration, and Transfusion Exposure Risk in DEHP Versus Non-DEHP Blood Storage Systems: A Mechanistic and Quantitative Comparative Analysis

**DOI:** 10.3390/cells15141276

**Published:** 2026-07-16

**Authors:** Ludwig Rayner Frontier Ramos, Wouter Van’t Hof, Quentin Brebant, Richard R. Gammon

**Affiliations:** 1 Medical Affairs, Macopharma USA, 3075 Breckinridge Blvd, Suite 405, Duluth, GA 30096, USA; 2 Cleveland Cord Blood Center, Cleveland, OH 44106, USA; wvanthof@clevelandcordblood.org; 3 Research and Development Department, Macopharma, 200 Chaussée Fernand Forest, 59200 Tourcoing, France; quentin.brebant@macopharma.com; 4 Department of Hematopathology, Moffitt Cancer Center & Research Institute, 12902 Magnolia Drive, Tampa, FL 33612, USA; richard.gammon@moffitt.org; 5 Department of Oncologic Services, University of South Florida, Tampa, FL 33612, USA

**Keywords:** platelet storage lesion, DEHP, migration of plasticizers, platelet storage system, biomaterial-cell interaction, platelet additive solution, modeling transfusion exposure

## Abstract

**Highlights:**

- Replacing di(2-ethylhexyl) phthalate (DEHP) with modern alternative plasticizers for platelet storage systems has the potential to not affect platelet properties or clinical effect while significantly reducing plasticizer migration and modeled patient exposure burden.
- DEHP found in whole-blood collection bags before platelet products are prepared enters platelet products with its major metabolite, mono-2-ethylhexyl phthalate (MEHP), posing a potential risk of exposure to recipients. This migration and the associated burden of exposure can be significantly decreased by eliminating DEHP in the collection system.
- The existing mechanistic data show that substituting DEHP with alternative plasticizers has not been shown to impair measured platelet function endpoints and significantly reduces patients’ potential exposure to plasticizer-derived compounds, making it possible to support further evaluation of phthalate-free blood storage systems.

**Abstract:**

The global transition away from di(2-ethylhexyl) phthalate (DEHP)-plasticized blood storage systems has raised important questions regarding platelet storage biology, transfusion performance, and recipient exposure to plasticizer-derived compounds. This systematic review evaluated platelet quality, plasticizer migration, and modeled transfusion exposure associated with DEHP-containing and DEHP-free platelet storage systems, including di(2-ethylhexyl) terephthalate (DEHT), diisononyl cyclohexane-1,2-dicarboxylate (DINCH), butyryl trihexyl citrate (BTHC), and tri(2-ethylhexyl) trimellitate (TOTM). English-language studies reporting quantitative platelet storage, migration, or exposure endpoints were identified through structured Google Scholar searches through 1 May 2025, supplemented by manual reference screening. Eligible studies were required to specify plasticizer composition and report quantitative biologic, migration, or exposure outcomes. Narrative reviews, opinion articles, and studies lacking extractable quantitative data were excluded. The risk of bias and the certainty of the evidence were assessed using adapted domain- and GRADE-based frameworks for mechanistic and preclinical studies. A random-effects meta-analysis was performed for day 7 platelet pH, while the remaining endpoints were synthesized descriptively. Thirty-three studies were included, comprising experimental platelet storage studies, biomaterial migration analyses, toxicologic investigations, and exposure-modeling studies. Platelet metabolic stability, including pH, glucose consumption, lactate accumulation, and mitochondrial membrane potential, was preserved across plasticizer systems. Meta-analysis demonstrated no significant difference in pooled day 7 platelet pH between DEHP and non-DEHP systems (mean difference +0.025; 95% confidence interval −0.081 to +0.131). Platelet aggregation and agonist-induced activation responses were maintained in DEHP-free systems. Selected activation and apoptotic markers, including CD62P expression and Annexin V binding, were reduced in fully DEHP-free systems, suggesting decreased storage-related membrane stress. Migration studies consistently demonstrated substantially greater DEHP leaching than DINCH and DEHT, resulting in higher modeled cumulative transfusion exposure, particularly among pediatric and neonatal recipients. Evidence further indicated that DEHP-containing whole-blood collection systems contribute to downstream contamination of platelet products with DEHP and mono(2-ethylhexyl) phthalate (MEHP). Interpretation is limited by heterogeneity in study design, storage platforms, assay methodologies, and the predominance of in vitro evidence. Overall, replacing DEHP with modern alternative plasticizers helps preserve transfusion-relevant platelet quality while potentially reducing plasticizer migration and modeled recipient exposure, thereby supporting the biologic and toxicologic rationale for transitioning to phthalate-free blood collection and storage systems.

## 1. Introduction

Platelet transfusion is an essential component of supportive care in hematology, oncology, emergency medicine, and transplantation. Although significant improvements have been made in donor screening, component processing, and storage, platelets are uniquely vulnerable to storage-induced injury, progressive activation, and functional loss during ex vivo preservation, a condition commonly known as the platelet storage lesion [1,2]. Compared with the broad biological characterization of platelet physiology and metabolism, the materials in which platelets are stored, especially those based on plasticized polyvinyl chloride, have traditionally received relatively little attention compared with platelet biology and functional integrity relative to red blood cells (RBCs). This gap has become increasingly relevant, as plasticizers may leach or migrate from collection and storage products into platelet products, thereby linking device composition to platelet quality and recipient exposure. Recent analytical research has reported distinct migration patterns of DEHP, DINCH, and DEHT among blood constituents and supports the role of biomaterial–blood interactions in transfusion systems [3].

Historically, the most common plasticizer employed in blood bags and related tubing has been DEHP because of its flexibility, durability, compatibility with PVC, and good processing characteristics [4,5]. Nevertheless, the growing body of toxicological, regulatory, and biological research has led to a global shift away from DEHP toward alternative plasticizers [4]. This transition raises the question in transfusion medicine of whether the chemical composition of the plasticizer used in platelet storage systems may affect platelet metabolic stability, platelet activation status, and subsequent transfusion performance. Moreover, since platelet concentrates can be made from whole-blood units collected using a DEHP-containing kit, exposure to plasticizers can occur before transfer into the final platelet storage container.

The early clinical trials of platelet storage were primarily focused on post-transfusion recovery and platelet survival. Significant randomized trials have shown that platelet additive solutions could replace a substantial proportion of plasma without compromising the measured clinical endpoints, without compromising corrected count increments or bleeding results [1]. The above clinical and observational research was further confirmed by subsequent research showing that platelets stored in additive solutions were less likely to cause allergic transfusion reactions, with no change in clinical efficacy, compared with platelets stored in plasma [2]. These studies collectively made platelet additive solutions an acceptable method of storage in clinical practice. Nonetheless, they did not address the potential biological effects of plasticizer migration from storage materials into platelet concentrates or how the collection set materials contributed to downstream contamination.

The toxicologic profile of DEHP has been extensively characterized. Since DEHP is not covalently attached to PVC, it can leach into lipid-rich biological fluids, where it is converted to biologically active monoesters [5,6]. European and North American regulatory bodies have constantly raised concern, largely based on in vitro and animal studies, regarding reproductive toxicity, endocrine disruption, and cumulative exposure, especially in groups that undergo repeated medical procedures [3,7]. As a result, DEHP has been categorized as a Substance of Very High Concern under the European Registration, Evaluation, Authorization, and Restriction of Chemicals (REACH) regulatory framework, and international regulatory bodies have encouraged the use of safer alternative plasticizers in medical devices where practicable [4,6,7,8]. These concerns are heightened in the context of transfusion by the possibility of repeat exposures and the vulnerability of pediatric and neonatal recipients.

In transfusion medicine, early experimental research revealed a quantifiable transfer of DEHP into platelet concentrates and plasma, as well as measurable systemic exposure in transfusion recipients. This was followed by further studies that applied these results to blood donors undergoing apheresis therapies and found DEHP metabolites in platelets and plasma after donation [9]. Although these studies did not attempt to examine platelet functions per se, they provided a valuable mechanistic basis for analyzing platelet storage biology from a biomaterial-exposure perspective. Importantly, they also suggest that exposure can occur at multiple workflow stages, including collection, processing, and storage.

Comparisons of DEHP-containing platelet storage systems and those without DEHP were facilitated by the development of alternative plasticizers, such as butyryl trihexyl citrate (BTHC), DINCH, tri(2-ethylhexyl) trimellitate (TOTM), and DEHT [9,10,11]. One of the early studies to establish that platelets stored in non-DEHP containers retained acceptable metabolic and aggregatory properties over 5 days of storage was by Snyder and colleagues, who overturned the long-held belief that platelets could not be preserved without DEHP [11]. Later studies reported preserved pH homeostasis, aggregation behavior, and platelet morphology in other plasticized storage systems [10]. Nevertheless, results vary across studies due to platform (apheresis vs. whole-blood-derived platelets), additive solution composition, and the specific device components containing DEHP.

Taken together, the current literature supports a conceptual model in which the composition of plasticizers, the chemistry of the additive solutions, and the duration of storage may influence platelet metabolic stability, platelet activation status, and platelet functional integrity. Nevertheless, evidence so far is sparse in toxicology, materials science, in vitro platelet biology, and clinical transfusion studies. This heterogeneity highlights the importance of an integrated, systematic assessment that considers platelet quality endpoints, plasticizer migration (including upstream sources), and modeled recipient exposure.

The review provides a systematic review of the literature comparing DEHP and DEHP-free platelet storage systems, combining platelet biological endpoints with data on biomaterial migration and modeled estimates of platelet-transfusion exposure. We hypothesized that modern alternative plasticizers could maintain platelet metabolic and functional quality when substituting for DEHP and minimize plasticizer migration and exposure to potential recipients. Unlike previous narrative reviews that focused primarily on toxicological concerns, this systematic review integrates mechanistic platelet quality parameters, plasticizer migration profiles, and quantitative exposure estimates. It evaluates the methodological certainty of the available evidence. This review also integrates platelet biology, plasticizer migration, quantitative exploratory exposure modeling, and risk-of-bias assessment.

This study combines platelet cellular physiology, biomaterial migration studies, and deterministic exposure simulations to offer a mechanistically informed framework for assessing plasticizer replacement in blood storage technologies. This strategy aligns with current priorities in transfusion medicine, focusing on the principles of safety-by-design and evidence-based assessment of devices [12,13,14]. The synthesis of available mechanistic evidence that DEHP-free systems do not impair platelet quality is thus crucial to scientific rigor, regulatory alignment, clinical decision-making, and the potential to reduce exposure to leachable compounds.

## 2. Materials and Methods

### 2.1. Study Design

**Figure 1:**
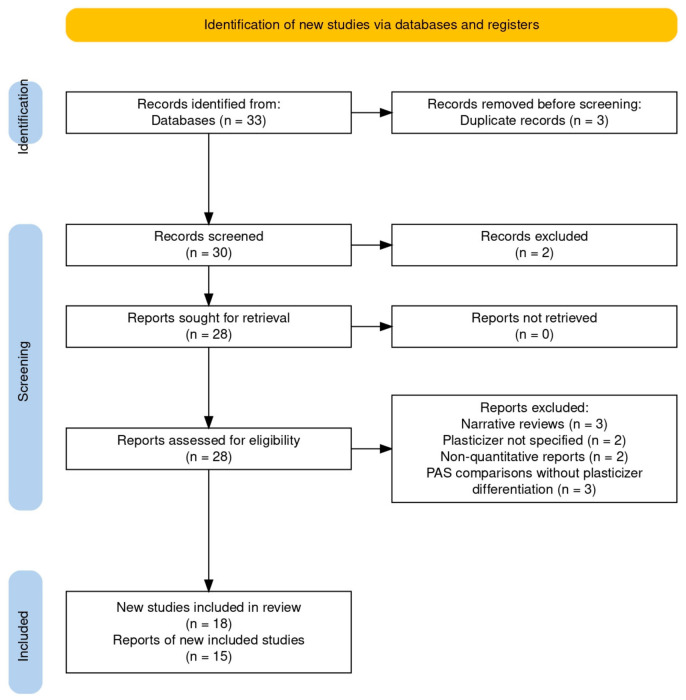
PRISMA flow diagram illustrating study identification, screening, eligibility, and inclusion.

This study was conducted as a systematic mechanistic evidence synthesis to evaluate platelet storage biology, biomaterial plasticizer migration, and modeled transfusion exposure associated with DEHP and non-DEHP blood storage systems. The review was performed in accordance with the Preferred Reporting Items for Systematic Reviews and Meta-Analyses (PRISMA) 2020 guidelines [15], and the study identification and selection process is summarized in Figure 1. A predefined analytical framework included three domains: (1) platelet metabolic and functional integrity in storage, (2) plasticizer migration into blood components in collection, processing, and storage, and (3) projected exposure of the recipient to transfusion in clinically relevant scenarios.

### 2.2. Information Sources and Search Strategy

Google Scholar (Google LLC, Mountain View, CA, USA) was used as the primary search platform because of its broad multidisciplinary indexing of the biomedical, biomaterials, toxicology, engineering, and transfusion medicine literature relevant to blood storage technologies. Structured searches were conducted through 1 May 2025 (no earliest date was set), using predefined Boolean search strategies, supplemented by targeted author searches and backward/forward citation screening. To verify the completeness of the search strategy, a confirmation search was subsequently performed in PubMed, maintained by the U.S. National Library of Medicine, Bethesda, MD, USA, using the same search concepts and eligibility criteria. This verification search confirmed the principal studies identified in the primary search and did not materially change the evidence synthesis.

Searches were limited to English-language publications in Google Scholar. The search strategy was developed to include studies that assessed platelet storage quality, plasticizer migration or leaching, and transfusion-related exploratory exposure modeling for DEHP and other plasticizers, such as DEHT, DINCH, BTHC, and TOTM. No formal review protocol was prepared.

Controlled vocabulary and free-text terms used in the search strategy included concepts related to platelet storage, plasticizer chemistry, biomaterial migration, and toxicological assessment. The search strategy combined terms related to platelet storage, plasticizers, platelet quality, biomaterial migration, transfusion exposure, and toxicology modeling. The following search terms and combinations were used: “platelet storage,” “platelet concentrate,” “platelet preservation,” “DEHP,” “di(2-ethylhexyl) phthalate,” “plasticizer,” “PVC,” “blood bag,” “DEHT,” “DINCH,” “BTHC,” “TOTM,” “platelet quality,” “metabolism,” “activation,” “aggregation,” “plasticizer migration,” “leaching,” “extractables,” “blood bags,” “plasma,” “red blood cells,” “transfusion exposure,” “platelet transfusion,” “blood transfusion,” “DEHP exposure,” “phthalate exposure,” “modeling,” “toxicology,” “risk assessment,” “platelet additive solution,” “PAS,” “platelet storage lesion,” “mitochondrial function,” “CD62P,” and “Annexin V.”

Author-name searches and backward/forward citation tracking (e.g., Thelliez plasticizer migration blood bags, Lagerberg platelet DINCH) were used to identify relevant mechanistic and analytical studies. Manual screening of the reference lists of articles included was conducted to identify additional eligible studies. Relevance ranking was used to filter the first 200 results of each query.

The certainty of the evidence was assessed using an adapted GRADE method for preclinical and mechanistic studies. Certainty was assessed using adapted GRADE domains, including risk of bias, inconsistency, indirectness, imprecision, and publication bias. The authors independently rated and reached consensus on the final ratings. Since the evidence base was mainly in vitro and analytical studies, indirectness and imprecision were anticipated and systematically factored into the grading process.

### 2.3. Eligibility Criteria

Eligible studies were required to clearly describe the plasticizer content of the blood storage systems and provide quantitative endpoints on platelet storage biology, biomaterial migration, or transfusion exploratory exposure modeling. The exclusion criteria included: narrative reviews and opinion articles; failure to specify the plasticizer composition; lack of quantitative, extractable data; and clinical transfusion results only, with no platelet storage results.

### 2.4. Study Selection Process

Search results were filtered at the title, abstract, and full-text levels and against predefined eligibility criteria. Publications and other reports on the same dataset were combined, and a full-text evaluation was conducted. A structured form was used to screen and assess eligibility, ensuring uniform application of the criteria. In cases where several publications reported on overlapping datasets, the most complete report was retained and complemented with distinct endpoints from companion reports. The study’s characteristics are presented in Supplementary Table S1.

Study selection was performed by the corresponding author using predefined eligibility criteria, with subsequent review and consensus among the co-authors. Because independent duplicate screening was not performed, inter-reviewer agreement statistics (e.g., Cohen’s κ) were not calculated. Thirty-three publications met the eligibility criteria and were included in the evidence synthesis. In addition to the main database search, which was performed until 1 May 2025, 15 additional articles were identified during the full-text review period through manual reference screening and author searches. These studies met all predetermined eligibility criteria and were included in the final evidence synthesis. This additional identification procedure accounts for the discrepancy between the number of studies represented in the PRISMA flow diagram and the number of included studies (*n* = 33). The PRISMA flow diagram (Figure 1) summarizes the study selection process.

### 2.5. Data Items and Study Characteristics

Included records were categorized as quantitative experimental/migration/exposure studies or contextual mechanistic/regulatory sources.

Extracted data items included were:

- Preparation technique of platelets (apheresis vs. whole-blood-derived);
- Storage time and temperature;
- Composition of plasticizers and plasticizing device (collection set, storage container, tubing);
- Assay procedure (instrument, agonist concentrations to activate/aggregate);
- Quantitative endpoint values;
- Sample size and estimates of variance;
- Type of additive solutions and plasma fraction.

The categories of sample sizes were pragmatically determined according to the independent experimental unit or paired observations: small (*n* < 10), moderate (*n* = 10–30), and large *(n* > 30). Table 1 summarizes the studies’ characteristics and classifies the evidence.

Study characteristics and evidence categories are summarized in Table 1.

### 2.6. Data Extraction and Standardization

Text, tables, and additional literature were analyzed, and quantitative data were extracted and entered into structured evidence matrices. Endpoints were converted to common units where necessary, and storage time points were specified to enable comparison of results across studies, with special attention to day 7 measurements.

### 2.7. Risk of Bias and Evidence Classification

Since the studies included are mechanistic and heterogeneous, formal risk-of-bias tools were not used. Rather, studies were classified according to methodological relevance and reporting rigor across prespecified domains: (i) clarity of plasticizer identification and device components, (ii) comparability of storage conditions and solutions, (iii) use of paired/controlled designs, (iv) independence of experimental units, (v) assay validation and endpoint definitions, and (vi) completeness of reporting (*n*, variance, time points):

- High-weight evidence: controlled comparative platelet storage studies reporting quantitative endpoints.
- Moderate-weight evidence: mechanistic, migration, or exposure studies that do not involve a direct comparison.
- Contextual evidence: the regulatory/toxicologic literature.

High-weight evidence comprised controlled comparative platelet storage studies that directly evaluated DEHP and non-DEHP blood storage systems and reported quantitative platelet quality outcomes. Moderate-weight evidence included mechanistic, analytical, migration, and exposure studies that contributed important biological or toxicological information but did not directly compare storage systems. Contextual evidence consisted of regulatory and toxicological publications that informed the interpretation of the findings but were not considered direct comparative evidence. This hierarchy was established a priori to reflect the relevance of each evidence type to the review’s primary objectives and to ensure a transparent and consistent interpretation of the available literature.

**Figure 2:**
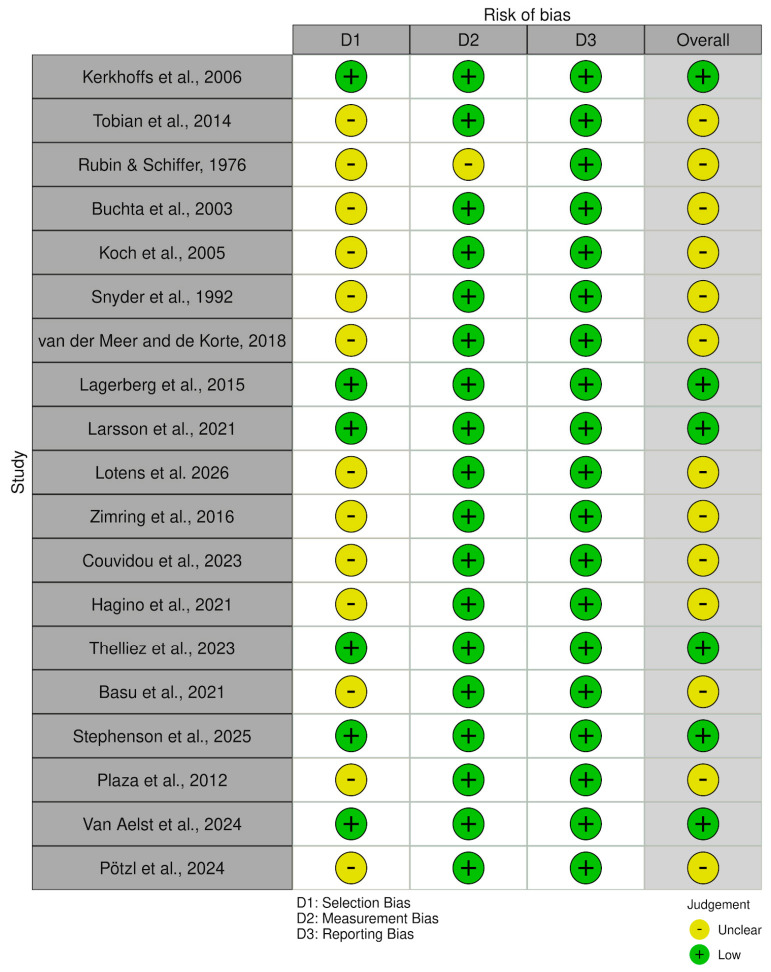
Risk of bias was assessed across three domains: D1, selection bias; D2, measurement bias; and D3, reporting bias. The overall judgement is shown in the final column. Green circles with a plus sign indicate low risk of bias, whereas yellow circles with a minus sign indicate unclear risk of bias. The studies assessed were Kerkhoffs et al., 2006 [1]; Tobian et al., 2014 [2]; Rubin and Schiffer, 1976 [6]; Buchta et al., 2003 [25]; Koch et al., 2005 [19]; Snyder et al., 1992 [11]; van der Meer and de Korte, 2018 [26]; Lagerberg et al., 2015 [9]; Larsson et al., 2021 [10]; Lotens et al., 2026 [23]; Zimring et al., 2016 [18]; Couvidou et al., 2023 [27]; Hagino et al., 2021 [28]; Thelliez et al., 2023 [3]; Basu et al., 2021 [12]; Stephenson et al., 2025 [13]; Plaza et al., 2012 [29]; Van Aelst et al., 2024 [16]; and Pötzl et al., 2024 [24].

**Figure 3:**
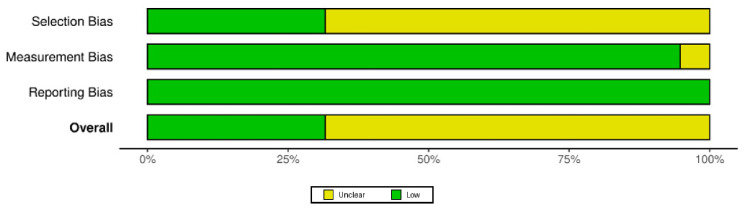
Traffic-light summary graph summarizing domain-level risk-of-bias assessments across included studies.

Table 2 summarizes the adapted risk-of-bias assessments across included studies, while Figure 2 and Figure 3 provide complementary visual representations of the domain-level risk-of-bias evaluation using traffic-light plots and summary graphs. Table 3 presents the adapted GRADE certainty-of-evidence analysis for the major outcome domains assessed in this review. Together, these frameworks enabled systematic incorporation of mechanistic, analytical, and translational evidence while maintaining methodological transparency and consistency in interpretation alongside the study-level evidence classification summarized in Table 1.

### 2.8. Outcome Measures and Harmonization

Endpoints were aligned to standardized areas of analysis:

▪ Metabolic status/stability (pH, glucose, lactate);
▪ Mitochondrial activity (mitochondrial inner membrane potential ΔΨm);
▪ Annexin V binding and apoptosis (CD62P, Annexin V);
▪ Functional responsiveness (PAC-1 binding, aggregometry);
▪ Plasticizer migration (normalized concentrations);
▪ Modeled exposure (estimates of the dose to the recipient).

The main anchors of the mechanism were the platelet metabolic stability and activation cues.

### 2.9. Synthesis Methods

#### 2.9.1. Quantitative Synthesis (Meta-Analysis)

**Figure 4:**
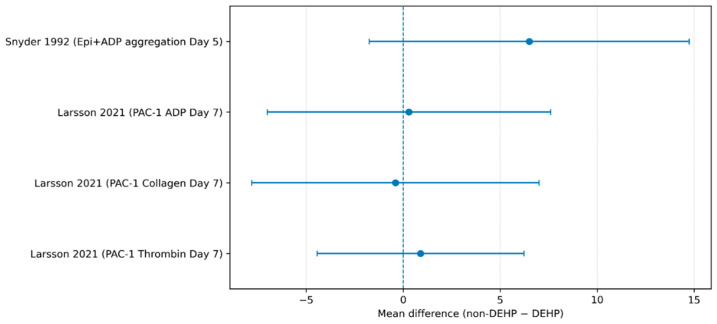
Study-level estimates of platelet aggregation and activation responses in DEHP versus non-DEHP storage systems. Mean differences were calculated as non-DEHP minus DEHP. Blue dots represent study-level point estimates, and horizontal lines represent the corresponding confidence intervals. The vertical dashed line at zero indicates no difference between DEHP and non-DEHP storage systems; confidence intervals crossing this line indicate no statistically significant difference between storage systems. The studies shown are Snyder et al. [11] and Larsson et al. [10]. DEHP, di(2-ethylhexyl) phthalate; PAC-1, activated GPIIb/IIIa binding antibody.

Random-effects meta-analysis (DerSimonian–Laird model) was used to analyze studies with similar endpoints and at least two studies. The only endpoint that met the pooling criteria was the day 7 platelet pH. Mean differences (non-DEHP -DEHP) were calculated. Cochrane’s Q test was used to assess statistical heterogeneity, and the I^2^ statistic was used to quantify the degree of heterogeneity, with I^2^ > 50% considered significant. Sensitivity analyses were not performed due to the limited poolable datasets. Study-level estimates of platelet aggregation and activation responses in DEHP versus non-DEHP storage systems are represented in Figure 4.

#### 2.9.2. Descriptive Synthesis

A descriptive analysis of endpoints that were not amenable to pooling was done. Platelet activation and aggregation were assessed based on the effect size, directionality, and overlap of the confidence intervals.

### 2.10. Plasticizer Migration and Exploratory Exposure Modeling

Migration data were converted to concentrations per unit of storage surface area and per unit volume of blood components. Descriptive comparisons were made between the migration patterns of DEHP and other plasticizers. Deterministic modeling was used to estimate recipient exposure based on migration coefficients, transfusion volume, storage surface area, and body weight. Adult, pediatric, and neonatal recipients were modeled. Exposures (daily and cumulative) were determined and adjusted by body weight.

### 2.11. Toxicologic Benchmarking

The exposure estimates in the models were expressed relative to known toxicologic reference values, such as the tolerable daily intake (TDI) and the no-observed-adverse-effect level (NOAEL). The exposure levels were reported as percentages of these levels.

### 2.12. Statistical Analysis

Continuous variables were summarized as the mean and standard deviation. The non-DEHP storage outcome value was compared with the DEHP storage outcome value to determine the mean difference. In situations where the data variance was known, confidence intervals were obtained. All calculations were performed in Microsoft Excel, Version 365 (Microsoft Corporation, Redmond, WA, USA). Estimation and heterogeneity tests were used to make statistical inferences consistent with mechanistic evidence synthesis.

### 2.13. Use of Generative Artificial Intelligence

ChatGPT, including the GPT-5.6 Thinking model (OpenAI, San Francisco, CA, USA), was used only to improve language, readability, and manuscript organization. All study design, literature screening, data extraction, analyses, interpretation, and final scientific content were performed and verified by the authors. All references and factual statements were checked against the original sources before submission. A Turnitin AI Writing Assessment (Turnitin LLC, Oakland, CA, USA) reported 0% AI-generated text.

## 3. Results

### 3.1. Study Selection and Evidence Base

The PRISMA flow diagram (Figure 1) summarizes the search and screening process.

### 3.2. Platelet Metabolic Stability in DEHP Versus Non-DEHP Storage Systems

**Figure 5:**
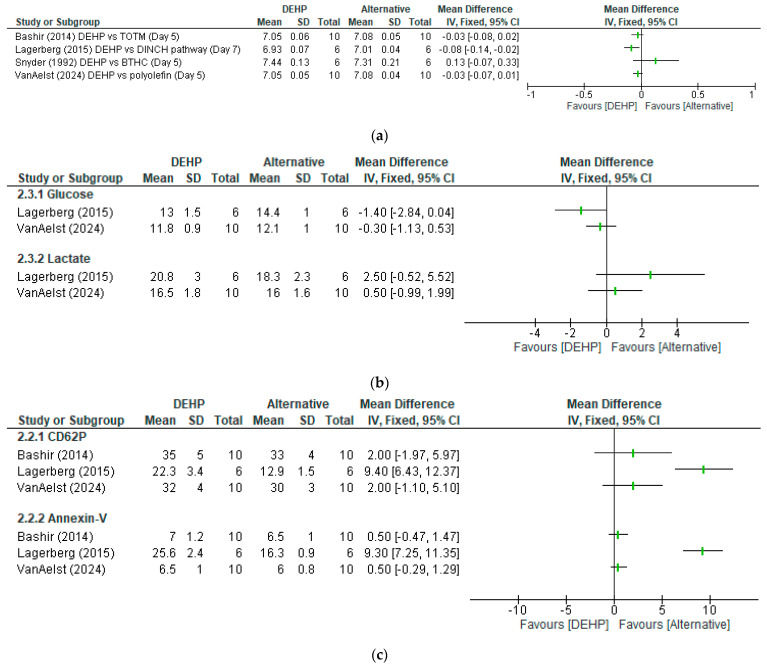

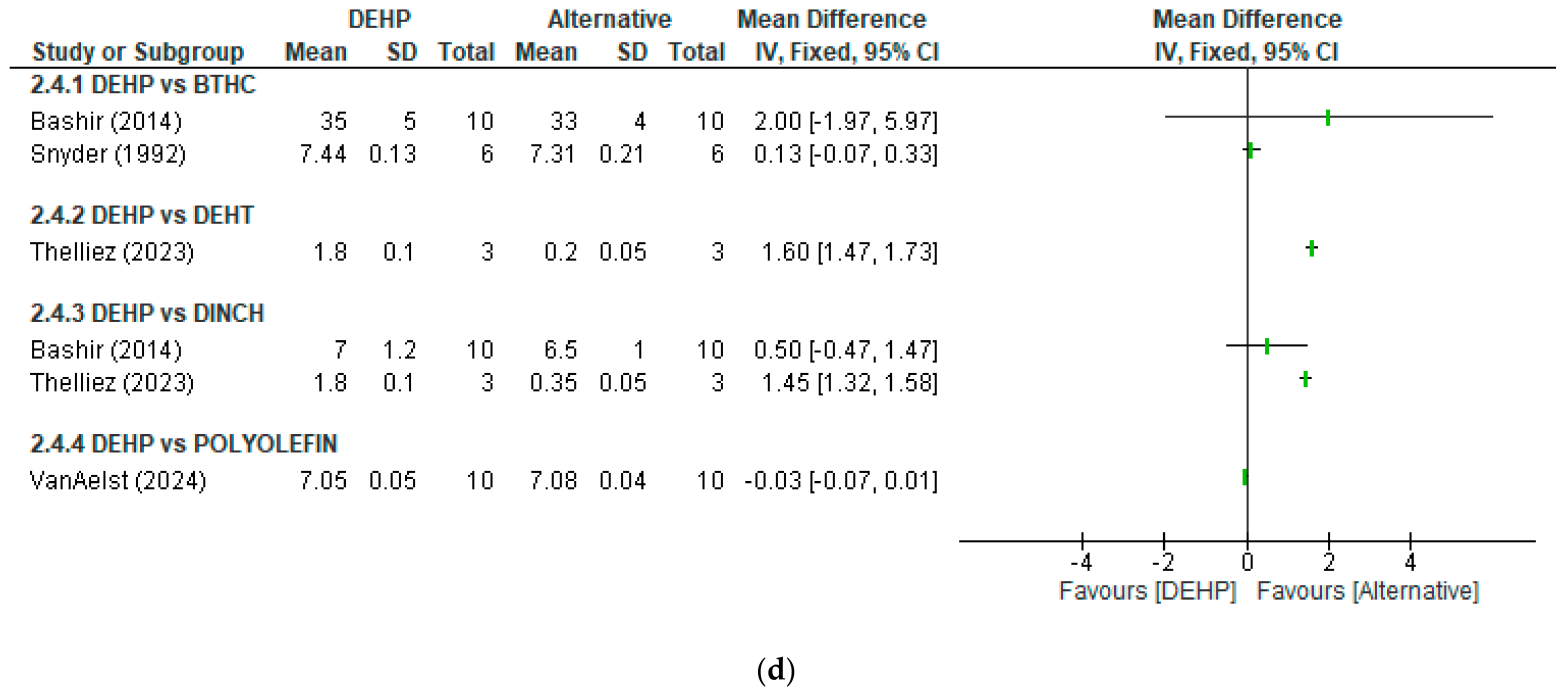
Integrated platelet storage and plasticizer migration endpoints in DEHP and non-DEHP systems. Mean differences were calculated as non-DEHP minus DEHP using a fixed-effect inverse-variance model and are shown with 95% confidence intervals. Green markers represent study-level mean differences, and horizontal lines represent the corresponding 95% confidence intervals. The vertical line at zero indicates no difference between DEHP and non-DEHP systems; confidence intervals crossing this line indicate no statistically significant difference between groups. Values to the left of zero favour DEHP, whereas values to the right of zero favour the alternative non-DEHP system. (**a**) Pooled mean difference in day 7 platelet pH. (**b**) Study-level metabolic stability endpoints. (**c**) Study-level differences in CD62P and Annexin V expression. (**d**) Plasticizer migration levels across DEHP, DINCH, and DEHT systems. Bashir et al., 2014 [22], Lagerberg et al., 2015 [9], Snyder et al., 1992 [11], Van Aelst et al., 2024 [16], Thelliez et al., 2023 [3].

Figure 5 summarizes integrated metabolic, activation, and migration endpoints in vitro. Table 4 summarizes comparative metabolic endpoints in vitro. In all studies, platelet metabolic homeostasis was maintained regardless of the plasticizer type, as evidenced by stable pH, glucose consumption, lactate production, and platelet-available biomarkers under standard storage conditions [9,11,16].

Mean differences were calculated as non-DEHP minus DEHP where applicable. Confidence intervals that cross zero indicate no statistically significant difference. DEHP, di(2-ethylhexyl) phthalate; DEHT, di(2-ethylhexyl) terephthalate; DINCH, diisononyl cyclohexane-1,2-dicarboxylate.

#### 3.2.1. pH Stability

Across comparative studies, terminal platelet pH remained comparable between DEHP and non-DEHP storage systems. The detailed study-level values are summarized in Table 4.

#### 3.2.2. Random-Effects Meta-Analysis at Day 7

The in vitro terminal storage pH of day 7 was the only endpoint considered for exploratory pooling. A random-effects meta-analysis using the Der Simonian–Lairdvitro approach showed a pooled mean difference (non-DEHP–DEHP) of +0.025 units of pH (95% CI, −0.081 to +0.131), which is not statistically significant. The results of the pooled meta-analysis are summarized in Table 5 and Figure 5a.

Between-study heterogeneity was high (I^2^ = 83.1%); this heterogeneity may reflect differences in platelet source, additive solution, and storage configuration [9,11]. The heterogeneity estimates should be interpreted with caution, given that there are only 2 studies (k = 2). However, the trend and magnitude of the effect estimate were uniform across the plasticizer systems, as indicated by overlapping terminal pH values (Figure 5a).

#### 3.2.3. Glycolytic Metabolism

The in vitro glucose consumption and lactate profiles in different plasticizer systems were similar (Figure 5b). Day 5 glucose levels were around 11.8 ± 0.9 mmol/L and 12.1 ± 1.0 mmol/L in DEHP and non-DEHP systems, respectively, and lactate levels were 16.5 ± 1.8 mmol/L and 16.0 ± 1.6 mmol/L [16].

In pediatric platelet cultures, there were minor differences. Glucose was slightly higher and lactate slightly lower in fully DEHP-free storage systems on day 7. Still, these changes were small relative to within-study variability and did not indicate changes in metabolic kinetics [9]. In general, glycolytic activity did not deteriorate across any storage platform, and no directional effect was observed. That could be attributed to plasticizer composition (Figure 5b).

#### 3.2.4. Mitochondrial Membrane Potential (ΔΨm)

**Figure 6:**
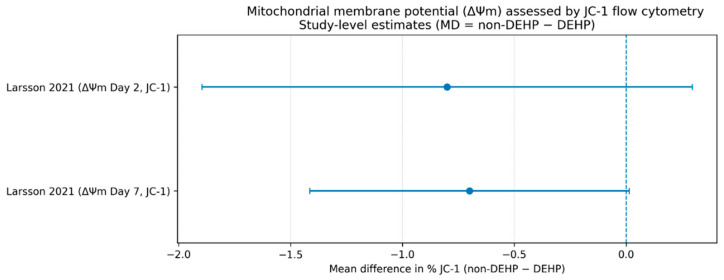
Study-level estimates of mitochondrial membrane potential (ΔΨm) in DEHP versus non-DEHP platelet storage systems. Mean differences were calculated as non-DEHP minus DEHP. Blue dots represent study-level point estimates, and horizontal lines represent the corresponding confidence intervals. The vertical dashed line at zero indicates no difference between DEHP and non-DEHP storage systems; confidence intervals crossing this line indicate no statistically significant difference between storage systems. The study shown is Larsson et al. [10].

Mitochondrial membrane potential (ΔΨm), measured by JC-1 flow cytometry, was used as a marker of platelet bioenergetic integrity and of stress associated with early storage. Study-level comparisons at day 2 and day 7 are summarized in Figure 6 and show preserved mitochondrial polarization in non-DEHP systems relative to DEHP comparators.

These results suggest that mitochondrial activity does not change during storage in DEHP-free systems and that metabolic profiles are comparable at the subcellular level under the conditions evaluated. This finding is consistent with broader metabolomic data indicating that platelet biomarkers are associated with platelet viability and post-transfusion performance [18]. In all available data, no evidence of mitochondrial dysfunction due to plasticizer replacement was found.

### 3.3. Platelet Activation and Apoptotic Signaling

Figure 5c summarizes the in vitro platelet activation marker (CD62P) and senescence marker (Annexin V), as well as apoptosis endpoints. Although overall platelet viability was maintained across the plasticizer systems, there were modest differences in selected activation- and apoptosis-related markers.

Pediatric platelet storage systems with a fully DEHP-free system were found in vitro to have reduced day 7 platelet CD62P expression (12.9 ± 1.5% vs. 22.3 ± 3.4%). Annexin V binding (16.3 ± 0.9% vs. 25.6 ± 2.4%) was lower in the non-DEHP system than in the DEHP-exposed system [9]. These results suggest decreased platelet activation and phosphatidylserine externalization, which are correlated with the inhibition of storage-related membrane stress and apoptotic signaling pathways.

In contrast, PAS-based storage comparisons showed overlapping distributions of activation markers between the DEHP and non-DEHP systems, with no statistically significant differences [16]. These findings indicate endpoint-specific variability and show no increase in activation in DEHP-free storage systems.

Together, these results indicate that replacing plasticizers does not increase platelet activation during storage and, in certain preparations, can suppress indicators of platelet markers consistent with reduced activation/senescence pathways (Figure 5c). These results are consistent with the known correlations among platelet activity, membrane remodeling, and the formation of storage lesions [18,30].

### 3.4. Platelet Functional Responsiveness and Aggregation

Table 6 summarizes in vitro study-level endpoints for aggregation and PAC-1 activation. Because only a few studies reported standardized functional assays under similar conditions, quantitative pooling was not possible; the results are reported as descriptive study-level estimates.

#### 3.4.1. Aggregation Capacity

In the included study, light-transmission aggregometry was used to measure platelet aggregation in vitro after stimulation with the dual agonist epinephrine and adenosine diphosphate (Epi + ADP). The aggregation responses were maintained across plasticizer systems, with mean aggregation values of 68.4 ± 9.2 in DEHP containers and 66.7 ± 8.5 in non-DEHP systems [11].

The difference in means (non-DEHP–DEHP) was −1.7% (95% CI −10.5 to +7.1), and the confidence intervals included zero, meaning that there could be no statistically significant difference between the aggregation capacity of non-DEHP and DEHP after storage (Table 6 and Figure 4). These results suggest that the platelet-aggregation potential was preserved after storage of non-DEHP systems.

#### 3.4.2. Platelet Activation Signaling (PAC-1 Binding)

The activation of integrin by agonists was assessed in vitro by PAC-1 binding after stimulation with ADP, collagen, and thrombin. The response to activation was similar across all agonist conditions in DEHP and non-DEHP systems, with mean differences of +0.3 (ADP), −0.4 (collagen), and +0.9 (thrombin), and all 95% confidence intervals were close to zero [10].

Since PAC-1 endpoint reporting under standardized conditions was available only in one study, quantitative pooling was not possible. Findings are thus presented as descriptive estimates at the study level. Across all conditions, there was no statistically clear difference, indicating that agonist-induced platelet activation signaling appeared to be preserved (Table 6). In Table 7, the descriptive study-level effect estimates for PAC-1 activation markers in DEHP versus non-DEHP platelet storage systems are described.

#### 3.4.3. Summary of Functional Endpoints

In both aggregation and activation assays, the effect estimates were small, with overlapping confidence intervals, and platelet functional responsiveness in non-DEHP storage systems was preserved. The results are consistent with the broader evidence suggesting that platelet functional performance depends primarily on storage conditions, additive solution composition, and metabolic preservation, rather than on plasticizer chemistry [26,29].

### 3.5. Plasticizer Migration and Modeled Transfusion Exposure

Comparative analytical studies demonstrated substantially greater in vitro migration of DEHP than of DINCH and DEHT across platelet and plasma storage systems, supporting the differential exposure profiles observed between plasticizer classes (Figure 5d). Plasticizers do not form covalent bonds with polyvinyl chloride matrices and thus partition into blood components upon contact, with the rate of migration determined by plasticizer chemistry, storage, and component-processing pathways [3]. The platelet concentrates showed the highest early migration signal, a result attributable to their lipid-rich composition and high surface-area exposure during storage.

On dday 1 of storage, in vitro platelet concentrates had measurable plasticizer concentrations, with equivalent concentrations of 1.80 µg/dm^2^/mL DEHP, compared with 0.30–0.40 µg/dm^2^/mL DINCH and 0.15–0.25 µg/dm^2^/mL. In labile blood products, DEHP migration was consistently higher in available datasets, with an exposure approximately 5.0- to 8.5-fold higher than DINCH and DEHT, respectively [3]. These variations were repeatable across matrices and storage conditions, and a consistent order of plasticizer migration was observed, with DEHP the most migratory, followed by DINCH and then DEHT.

This migration gradient reflects differences in physicochemical characteristics, such as lipophilicity, molecular structure, and affinity for biological matrices. DEHP is a highly lipophilic phthalate that exhibits high partitioning into lipid-rich matrices, such as plasma and platelet suspensions. Conversely, other plasticizers, such as DINCH and DEHT, are less leachable and less diffusive within blood components [3]. The ability of structural differences, such as the terephthalate structure of DEHT, to further reduce its migration relative to DEHP also contributes to the consistently low concentrations observed across all storage systems [3].

Exposure of platelets to plasticizers is cumulative and reflects the combined effects of multiple steps in the blood-processing chain, including whole-blood collection, component separation, processing, and final storage. The final concentration of the platelet product plasticizer is modulated by the contact surface area, exposure duration, and dilution effects during component preparation [3]. In platelet systems using whole blood, platelets are exposed to DEHP upstream through collection bags and processing components, potentially contributing to a downstream plasticizer load, even though end-use storage containers are free of the plasticizer.

In addition to the parent compounds, plasticizer breakdown products contribute to total exposure. Metabolites/degradation products, such as mono-2-ethylhexyl phthalate, monoisononyl cyclohexane dicarboxylate, and mono-2-ethylhexyl terephthalate, can be detected. In vitro during storage. They can account for approximately 17–47% of the compound’s equivalent plasticizer concentration, depending on the storage conditions and the compound [3]. The calculation of equivalent concentration, including these metabolites, provides a more comprehensive measure of the biological exposure of interest. Collectively, these results support the interpretation that the migration of plasticizers is a system effect governed by material characteristics, storage environments, and processing operations. The consistently higher migration of DEHP than that of other plasticizers provides a mechanistic foundation for greater transfusion-related exposure and for downstream integration of exploratory exposure modeling.

Table 8 presents data on migration as equivalent plasticizer concentrations (micrograms per dm.2/mL), divided by the storage surface area and the component volume. Among the tested blood components, platelet concentrates have the greatest early migration signal. On dday 1, the concentration of DEHP is about 1.80 micrograms per dm.2 per mL, compared with 0.30 to 0.40 for DINCH and 0.15 to 0.25 for DEHT, forming a consistent migration pattern, with DEHP highest, followed by DINCH and then DEHT [3]. Migration values are experimentally measured under various storage conditions and serve as input to downstream exploratory exposure modeling. The reported values represent the combined contributions from the collection, processing, and storage components and are consistent with the system-level plasticizer exposure.

**Figure 7:**
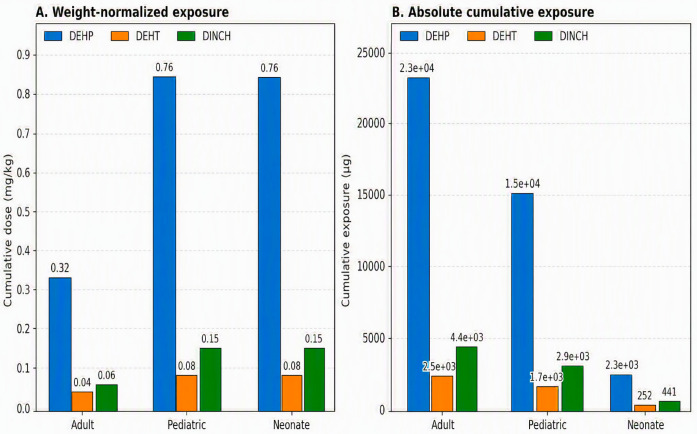
Exploratory modeling of cumulative plasticizer exposure across DEHP and non-DEHP platelet storage systems. Panel (**A**) shows weight-normalized cumulative exposure expressed as cumulative dose in mg/kg. Panel (**B**) shows absolute cumulative exposure expressed in µg. Estimates were calculated using migration coefficients derived from comparative plasticizer migration studies and deterministic transfusion assumptions. These projections are exploratory exposure estimates based on in vitro migration data and do not represent measured in vivo toxicologic dose. DEHP, di(2-ethylhexyl) phthalate; DEHT, di(2-ethylhexyl) terephthalate; DINCH, diisononyl cyclohexane-1,2-dicarboxylate.

Exploratory modeled cumulative exposure estimates were higher for DEHP-containing systems than for DEHT- and DINCH-based alternatives across adult, pediatric, and neonatal scenarios (Figure 7). Weight-normalized exposure burden was highest in neonatal recipients weighing 3 kg who received 30 mL/day platelet transfusions, reflecting the amplified effect of body mass scaling in low-weight populations (Table 9).

Exploratory cumulative exposure estimates derived from migration data are summarized in Table 9 and Figure 7. Migration coefficients, transfusion volume, storage-container surface area, and recipient body weight were used in exposure modeling to determine cumulative transfusion exposure under clinically relevant transfusion conditions.

Across all modeled scenarios, cumulative exposure followed the hierarchy DEHP > DINCH > DEHT. Weight-normalized exposure was highest in pediatric and neonatal scenarios because of body-mass scaling (Table 9).

In all simulated transfusion scenarios, the cumulative exposure to the plasticizer followed a steady hierarchy, with DEHP higher than DINCH and, in turn, higher than DEHT, consistent with experimentally determined migration gradients [3]. The exposure was weight-normalized and increased as body mass decreased, indicating overexposure in pediatric and neonatal groups.

Stacked bar projections illustrate cumulative plasticizer exposure derived from transfusion of red blood cells, plasma, and platelet concentrates stored in DEHP-, DINCH-, and DEHT-plasticized systems across adult, pediatric, and neonatal recipient scenarios. Component contributions are displayed as stacked segments representing RBC concentrates (bottom), plasma (middle), and platelet concentrates (top). Panel A depicts weight-normalized exposure (mg/kg) calculated using surface-area-normalized migration coefficients and a standardized 6 dm^2^ container contact area. The dashed horizontal line denotes the tolerable daily intake (TDI) threshold for DEHP (0.05 mg/kg/day). Panel B presents a relative exposure index derived from the product of migration and transfused volume, without surface-area normalization.

**Figure 8:**
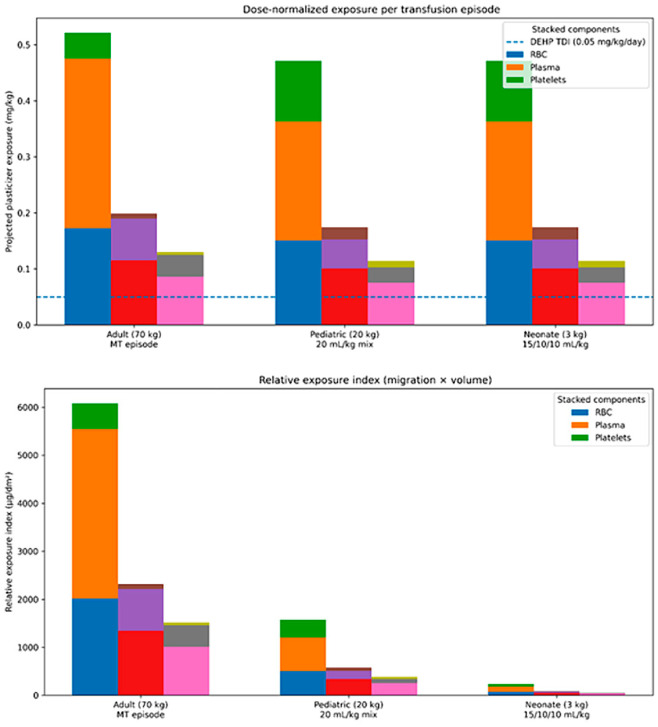
Exploratory modeling of cumulative plasticizer exposure from multi-component transfusion scenarios across DEHP and non-DEHP storage systems. Stacked bars represent estimated exposure contributions from RBC concentrates, plasma, and platelet concentrates. Panel A shows weight-normalized exposure in mg/kg. Panel B shows relative exposure index values based on migration and transfused volume. Estimates are exploratory and derived from in vitro migration data, not from measured in vivo exposure. RBC, red blood cell; DEHP, di(2-ethylhexyl) phthalate; DEHT, di(2-ethylhexyl) terephthalate; DINCH, diisononyl cyclohexane-1,2-dicarboxylate.

Figure 7 and Figure 8 show cumulative exposure to plasticizers, using stacked bar projections derived from transfusions of red blood cells, plasma, and platelet concentrates in adult, pediatric, and neonatal settings. The components’ contributions are represented as stacked segments, with red blood cell concentrate at the bottom, plasma in the middle, and platelet concentrates at the top, each indicating its contribution to the total exposure.

The exposure in Panel A has been normalized by weight. and expressed in milligrams per kilogram, based on surface-area-normalized migration coefficients and a standardized contact area for a 6 dm^2^ container. A horizontal reference line with dashed ends indicates the tolerable daily intake of DEHP at 0.05 mg/kg/day, which can be used to interpret modeled exposure relative to toxicologic standards.

The relative exposure indices in panel B are based on the product of normalized surface-area migration and transfused volume. This representation provides a complementary comparison of exposure magnitudes across plasticizer systems, regardless of body-weight normalization.

In both panels, platelet concentrates contribute substantially to overall exposure across contexts, consistent with experimentally observed higher platelet migration coefficients. These projections indicate that platelet transfusion is a significant contributor to plasticizer exposure in multi-component transfusion approaches and provide a qualitative framework for correlating biomaterial migration with recipient exposure.

In general, combining migration kinetics with deterministic exploratory exposure modeling indicates that replacing DEHP with other plasticizers can significantly reduce cumulative exposure during transfusion without compromising platelet storage capacity. The findings can be used to support the mechanistic and quantitative shift toward phthalate-free blood storage systems.

## 4. Discussion

### 4.1. Principal Findings

Several experiments have used contemporary cellular and molecular tests to assess platelet quality under DEHP-free storage conditions. Lagerberg and colleagues conducted extensive in vitro studies of platelets stored in DEHP-free systems. They reported maintaining metabolic stability and decreased expression of activation markers, including CD62P, compared with reference systems containing DEHP [9]. These results implied that plasticizer replacement may preserve platelet viability influence selected activation pathways, and that DEHP-free systems are a promising alternative requiring continued clinical evaluation.

In vitro studies of platelet biomarkers have been used as a sensitive measure of storage-associated stress at the subcellular level. Platelet bioenergetic competence is assessed by mitochondrial membrane potential, typically measured by the JC-1 flow cytometry assay, which tends to decrease before platelet metabolic failure occurs [23]. Comparative in vitro studies of DEHP use and DEHP-free storage systems have shown that mitochondrial membrane potential is maintained during storage, indicating that other plasticizers do not affect mitochondrial activity [9,10]. The observation is mechanistically pertinent, as phosphatidylserine exposure, microparticle formation, and increased platelet clearance have been attributed to mitochondrial depolarization [31].

In vitro functional testing also provides evidence supporting the biological similarity of DEHP-free storage platforms. Light transmission aggregometry and activation-dependent binding of the PAC-1 monoclonal antibody aggregation responses have generally been reported to be similar in DEHP-free and DEHP-containing storage systems after stimulation with ADP, collagen, or thrombin [10,11]. Taken together, these observations indicate that the mechanical and chemical characteristics of alternative plasticizers can be sufficient to maintain platelet viability without causing any detectable functional impairment.

In addition to single experimental observations, systems biology and metabolomic techniques have begun to link metabolic signatures associated with storage to post-transfusion platelet functionality. Zimring and colleagues reported that, in vitro, platelet metabolic pathways observed in stored platelets were associated with platelet recovery and survival in vivo, suggesting that the subtle metabolic changes that accumulate during storage are of translational relevance [18]. These data underscore the importance of considering storage materials and solutions as joint determinants of platelet biology.

This mechanistic, systematic synthesis shows that replacing DEHP with modern alternative plasticizers in platelet storage devices does not disrupt key indices of platelet metabolic stability, platelet activation responsiveness, or platelet functional aggregation capacity. These results show that the metabolic conditions necessary to maintain platelet viability, the mitochondrial membrane potential (Δψm), and functional platelet responsiveness are not significantly altered by plasticizer replacement, despite modest variations in the responses of some activation and senescence markers to plasticizers during ex vivo storage.

### 4.2. Biological Interpretation and Mechanistic Plausibility

DEHP is a lipophilic plasticizer that is not covalently bound to polyvinyl chloride matrices and can migrate into lipid-containing biological fluids [3,6]. After release, DEHP and its metabolites can affect cellular membranes, modifying membrane fluidity, lipid microdomain formation, and signaling pathways that mediate platelet activation and vesiculation [3,11,19]. Toxicologic experiments performed in vitro have shown that phthalate metabolites, including mono-2-ethylhexyl phthalate, can disrupt cellular signaling mechanisms and mitochondrial activity, a mechanistic explanation for biomaterial-induced physiological changes in platelets [32].

Analytical migration studies show a consistent rank order of plasticizer leachability among blood components, with DEHP showing significantly higher release than other plasticizers, such as DINCH or DEHT [3]. Platelet concentrates exhibit the highest early-migration signal among the components assessed, further supporting the biological significance of the platelet-plastic interface. The reduced exposure to membrane-active plasticizers in DEHP-free systems is also likely associated with lower CD62P expression and Annexin V binding observed in most storage models [9,16].

### 4.3. Translational Relevance: Activation Phenotypes and Expected In Vivo Performance

Platelet activation during storage is a known predictor of post-transfusion recovery and survival, as platelets that are prematurely activated or undergo apoptosis are more rapidly cleared by the reticuloendothelial system [30,31,33]. The recognition of macrophages and the reduction in platelet survival after transfusion have been linked to the expression of P-selectin (CD62P), the exposure of phosphatidylserine, and integrin activation signaling [27,28,30,31,33]. These mechanisms are important biological elements of the platelet storage lesion and contribute to transfusion effectiveness.

In that regard, the lack of greater activation signaling of non-DEHP storage systems is also a clinically relevant observation. The activation profiles and aggregation responses of PAC-1 are maintained across the plasticizer systems [10,11], suggesting that platelet responses to physiologic agonists were not clearly altered by plasticizer replacement.

In addition, the directionality of the activation results, along with multiple comparative datasets, suggests that removing exposure to phthalates could reduce, rather than increase, storage-induced membrane stress. This finding is consistent with general platelet storage articles, which show that additive solutions, the materials used to store the product, and metabolic preservation strategies all affect platelet quality and post-transfusion outcomes [1,5,26].

Metabolomic studies also suggest that minor metabolic changes observed during storage may be associated with post-transfusion platelet recovery and survival, indicating the biological implications of cellular storage signatures [18].

### 4.4. Plasticizer Migration and Cumulative Exposure Risk

Although platelet biological performance did not differ significantly between the plasticizer systems, migration analyses revealed that there may be substantial differences in platelet exposure to various plasticizers. In vitro studies showed that DEHP was found to migrate into blood components at higher levels than those of other plasticizers (e.g., DINCH and DEHT) [3]. Moreover, in platelet concentrates made from whole blood, exposure to DEHP may occur even before storage through contact with the primary whole-blood collection bag and processing materials (e.g., anticoagulant solution, transfer tubing, pooling sets, and intermediate containers) used during component preparation. In this way, platelet concentrate contamination could indicate cumulative workflow exposure to storage components, and eliminating DEHP from whole-blood collection kits is likely to reduce carryover of DEHP/MEHP into platelet concentrates.

The results are consistent with previous studies, which have shown the transfer of DEHP and its metabolites into blood components and their eventual presence in transfusion recipients and in donors exposed to plasticized medical devices [6,19,25].

Cumulative transfusion exposure, when used in deterministic exploratory exposure modeling, followed a consistent hierarchy of DEHP > DINCH > DEHT. Especially high weight-normalized exposure burdens were observed in pediatric and neonatal recipients due to decreased body mass and relatively higher transfusion volumes. Even though modeled exposures are below toxicologic thresholds, repeated transfusions in transfusion-dependent patients could result in a substantial cumulative plasticizer burden in populations chronically exposed to transfusions and have an effect on clinical outcomes. Therefore, the key benefit of removing DEHP is not enhanced platelet function but decreased patient exposure to plasticizers with known toxicologic profiles. Importantly, this exposure-reduction advantage is optimized when DEHP is eliminated not only from platelet storage vessels but also from upstream elements of the entire blood collection and processing, which together contribute to cumulative leachable exposure.

### 4.5. Regulatory and Manufacturing Transition Implications

The findings of this synthesis may have significant implications for the regulatory assessment of next-generation blood storage systems. DEHP has been classified as a substance of very great concern under the European REACH regulatory framework because it has reproductive organ toxicity and it has endocrine-disrupting potential [21,34]. Concurrently, the U.S. Food and Drug Administration has issued safety communications indicating potential risks of DEHP exposure among vulnerable patients.

In this policy context, the current synthesis provides mechanistic evidence suggesting that replacing DEHP with other plasticizers does not clearly impair measured metabolic stability, activation responsiveness, or aggregation endpoints. Meanwhile, migration studies indicate significantly less plasticizer transfer when modeled recipient exposure is DEHP-free. Given the potential for exposure at any point in the component-manufacturing process, a phthalate-free conversion should be considered at the kit stage (collection, processing, and storage), not only at the final platelet storage vessel.

Notably, the transfusion literature suggests that metabolic preservation, the composition of additive solutions, and storage conditions may be key determinants of platelet storage performance, rather than the plasticizer chemistry of storage containers themselves [1,2,12,13,15]. The overlap between retained platelet quality and decreased toxicologic exposure thus supports consideration of transition to phthalate-free blood storage technologies. In manufacturing terms, these data indicate that DEHP should be considered for removal from whole-blood primary bags and processing sets during the production of whole-blood-derived platelets, since upstream DEHP could be transferred to downstream platelet concentrates.

### 4.6. Implications and Future Directions

Prospective comparative studies that include harmonized activation panels, mitochondrial assays, and in vivo recovery endpoints across plasticizer systems should be the focus of future research. These would be enhanced by integrating leachable quantification with platelet quality endpoints to strengthen inference of biomaterial exposure to the biology of platelet storage lesions. Experiments that directly isolate upstream (collection/processing) versus downstream (storage) sources of leachable, especially in whole-blood-based platelet workflows, would further help explain where DEHP-free substitution is most likely to offer an exposure-reduction benefit.

Special attention should be given to populations such as neonatal and chronically transfused populations, where the burdens of cumulative exposure have the potential to be most significant. There will also be a need to continue harmonizing practices in the manufacturing of transfusion products, in light of evolving global standards for device use, to ensure that changes in platelet-storage biomaterials do not affect platelet availability or transfusion efficacy [25,27,28,33]. It should be noted that studies of platelet recovery/survival/transfusion outcomes remain limited.

### 4.7. Study Limitations

Several important limitations should be noted. Although Google Scholar was used as the primary search platform because of its broad multidisciplinary coverage, reliance on a single primary database and the absence of prospective protocol registration remain methodological limitations that should be considered when interpreting the findings. The predictive exposure and functional inference model is ecological in nature and uses summary estimates from in vitro studies rather than patient-level data. In turn, cross-study correlations between storage conditions, exposure to plasticizers, and transfusion efficacy must be viewed as hypothesis-generating rather than causal. The model aims to integrate mechanistic processes that bridge the gap between in vitro platelet quality parameters (e.g., pH stability, activation signaling, membrane integrity) and post-transfusion performance. Nonetheless, it cannot estimate clinical outcomes without patient-level covariates, immunologic status, or measures of transfusion burden. Moreover, most migration datasets provide component-wise quantification of plasticizers.

These datasets may not separate the relative contributions of individual workflow steps (primary whole-blood bag, processing set, and final storage container), thereby limiting step-specific attribution and supporting an overly conservative interpretation of source attribution in multi-component manufacturing pathways. It should also be noted that there is an absence of randomized clinical transfusion trials stratified by plasticizer. Some of the studies cited were conducted by industry, and even though the authors reviewed for bias, this may have affected some of the results. Lack of prospective registration may have limited methodological transparency. Another limitation was the small number of pooled studies. Sensitivity analyses and publication bias assessment were not performed due to the limited number of studies. The studies included variability from transfusion frequency, metabolism, storage conditions, and patient factors. Other limitations were that some of the models lacked external clinical validation and that randomized clinical trials comparing plasticizer systems were lacking.

## 5. Conclusions

This systematic multi-endpoint analysis of mostly in vitro studies and modeling indicates that DEHP-free platelet storage systems may be feasible and biologically plausible, capable of replacing DEHP-plasticized systems, maintaining basic indices of platelet storage quality, and reducing plasticizer migration and modeled recipient exposure burden. In comparative data, essential parameters of platelet metabolic integrity, such as pH stability, glycolytic activity, and mitochondrial membrane polarization, were all similar across plasticizer platforms, and there was no indication of increased storage-lesion formation in non-DEHP systems. Functional responsiveness, as measured by agonist-induced integrin activation and platelet aggregation assays, also suggested maintained signaling capacity and hemostasis-related functional responses following plasticizer substitution.

In contrast to the broadly similar metabolic endpoints, a directional pattern of platelet activation and apoptotic signaling was observed, as indicated by certain markers of platelet activation and apoptosis, with a preference for DEHP-free storage pathways. The decreased expression of CD62P and reduced Annexin V binding in fully phthalate-free systems may indicate inhibition of storage-related perturbations and senescence signaling in membranes during ex vivo preservation. Even though dataset and endpoint heterogeneity preclude mechanistic attribution, these findings are consistent with the hypothesis that reduced exposure to membrane-active plasticizers can preserve platelet quiescence during preservation.

Migration analyses of plasticizers provide a significant background of exposure for these cell observations. Across all tested blood component matrices, in vitro studies showed that DEHP was consistently more leachable than the other plasticizers (DINCH and DEHT), and platelet concentrates displayed the greatest initial migration signal. Notably, in the case of platelet concentrates prepared using whole blood, exposure can be upstream of the platelet storage container because the entire workflow (collection, component processing, and storage) may be contaminated by DEHP-containing primary whole blood bags and processing/transfer sets; thus, the measured contamination of platelet concentrates can be due to cumulative workflow exposure (collection, processing, and storage) and not solely to the storage bag. In modeled transfusion scenarios, cumulative exposure models showed the same hierarchy, with weight-normalized exposure burdens increasing substantially in pediatric and neonatal recipients due to body-mass scaling. These results demonstrate the necessity of considering biomaterial–cell contacts not only with respect to platelet storage functionality but also to the potential exposure of the recipient to leachable device materials.

Taken together, the preservation of platelet metabolic stability, retained functional responsiveness, decreased activation signaling, and reduced exposure burden during migration have the potential to affirm both the biological and translational feasibility of transitioning to phthalate-free platelet storage technologies. Notably, the results suggest that the substitution of plasticizers does not alter the core features of platelet storage biology but rather represents a materials-safety optimization. It is anticipated that this exposure-reduction benefit will be maximized when DEHP is eliminated not only in the final platelet storage vessel but also in upstream whole-blood collection and processing components that contribute to the cumulative leachable burden.

Overall, this paper illustrates the potential of combining platelet cellular physiology, biomaterial migration analysis, and exploratory exposure modeling to provide a mechanistically driven framework for evaluating emerging blood-storage technologies. With the current trend in transfusion medicine towards safer device materials and reduced exposure to potentially harmful leachables, the analysis suggests that DEHP replacement may be feasible without clear impairment of measured in vitro platelet storage endpoints. However, direct clinical efficacy data remain limited, making it a promising alternative requiring continued clinical evaluation. Future work should focus on step-resolved, leachable measurements to differentiate between collection/processing and storage contributions, especially for whole-blood-derived platelet workflows, and to coordinate platelet quality panels and in vivo recovery clinical endpoints. Future prospective studies that include harmonized platelet quality tests, in vivo recovery studies, and standardized monitoring of leachables may enhance the scientific framework that informs biomaterial innovation in blood component production.

## Figures and Tables

**Table 1:** Key evidence contributing to the assessment of DEHP and non-DEHP blood storage systems.

Study/Source	Evidence Category	Main Endpoint Domain	Design Relevance	Evidence Weight
Snyder et al., 1992 [11]	Platelet storage comparison	Metabolism, aggregation	Controlled paired container comparison	High
Lagerberg et al., 2015 [9]	Pediatric platelet storage	Activation, apoptosis, metabolism	Paired DEHP-exposed vs. fully DEHP-free comparison	High
Larsson et al., 2021 [10]	DEHT storage-system evaluation	Metabolism, activation, platelet quality	Controlled DEHT storage system evaluation	High
Van Aelst et al., 2024 [16]	PAS storage comparison	Metabolism, activation	Modern PAS platform comparison	High
Braathen et al., 2019 [17]	Platelet storage study	Storage metabolism, platelet function	Storage-condition evaluation	Moderate/indirect
Zimring et al., 2016 [18]	Metabolomics study	Platelet metabolic profile	Context for platelet recovery	Moderate/contextual
Koch et al., 2005 [19]	Plasticizer exposure study	DEHP metabolism	DEHP metabolite analysis	High
Thelliez et al., 2023 [3]	Plasticizer migration study	Biomaterial migration	Comparative plasticizer migration analysis	High
SCENIHR report, 2015 [4]	Regulatory toxicology	Exposure risk	Regulatory toxicology assessment	Moderate/contextual
FDA safety communication [20]	Regulatory safety assessment	Exposure risk	DEHP medical-device safety assessment	Moderate/contextual
ECHA REACH SVHC classification [21]	Regulatory classification	Exposure classification	REACH SVHC classification of DEHP	Moderate/contextual

Evidence weight reflects the relevance of each source to platelet storage quality, plasticizer migration, or transfusion-related exposure. Detailed methodological information, including sample size category and study-specific limitations, is provided in the Supplementary Material. PAS, platelet additive solution; DEHP, di(2-ethylhexyl) phthalate; DEHT, di(2-ethylhexyl) terephthalate; SVHC, substance of very great concern.

**Table 2:** Simplified methodological appraisal of key mechanistic and analytical studies.

Study	Study Role	Comparator Clarity	Reporting Completeness	Main Limitation	Overall Appraisal
Snyder et al., 1992 [11]	Platelet storage comparison	Clear	Moderate	Small paired sample size	Some concerns
Lagerberg et al., 2015 [9]	Pediatric platelet storage	Clear	Good	Small sample size and pediatric workflow specificity	Some concerns
Van Aelst et al., 2024 [16]	PAS-E storage comparison	Clear	Limited	Several graph-derived estimates	High concern
Bashir et al., 2014 [22]	Pediatric aliquot storage model	Indirect	Limited	Literature-based comparator and graph-derived values	High concern
Lotens et al., 2026 [23]	DEHT storage comparison	Clear	Moderate	Modest sample size and in vitro design	Some concerns
Thelliez et al., 2023 [3]	Plasticizer migration study	Clear	Good	Migration endpoints are indirect for platelet function	Some concerns
Pötzl et al., 2024 [24]	Exposure context	Indirect	Moderate	Limited direct relevance to platelet storage	Some concerns
Koch et al., 2005 [19]	Plasticizer exposure study	Indirect	Moderate	Indirect to platelet storage performance	Some concerns
Zimring et al., 2016 [18]	Platelet biology context	Not applicable	Moderate	No DEHP versus non-DEHP comparison	Contextual only

This simplified appraisal focuses on domains most relevant to the interpretation of platelet storage, blood-processing workflows, plasticizer migration, and transfusion-related exposure. Detailed domain-level judgments for sample size, assay objectivity, storage-platform confounding, and graph-derived estimates are provided in the Supplementary Material. PAS, platelet additive solution; DEHP, di(2-ethylhexyl) phthalate; DEHT, di(2-ethylhexyl) terephthalate.

**Table 3:** Certainty of evidence for DEHP versus non-DEHP platelet storage systems.

Outcome Domain	Evidence Base	Main Finding	Main Limitation	Overall Certainty
Platelet pH and metabolic stability	3–5 in vitro comparative studies	Terminal pH and glycolytic stability were broadly comparable between DEHP and non-DEHP systems.	Storage-platform heterogeneity and limited sample size	Low
Glucose consumption and lactate accumulation	2–3 in vitro comparative studies	Metabolic trajectories were comparable across plasticizer systems.	Limited number of studies	Low
Mitochondrial membrane potential	1–2 in vitro mechanistic studies	Mitochondrial polarization appeared preserved in non-DEHP systems.	Sparse evidence and indirectness	Very low
CD62P platelet activation	2 in vitro comparative studies	No increase in activation was observed; lower CD62P was reported in selected DEHP-free systems.	Endpoint and platform heterogeneity	Low
Annexin V binding	2 in vitro comparative studies	Lower Annexin V binding was reported in selected non-DEHP systems.	Limited number of studies	Low
PAC-1 functional responsiveness	1–2 in vitro functional studies	No evidence of impaired agonist-induced activation was observed in non-DEHP systems.	Few standardized datasets	Very low
Aggregation response	1–2 in vitro aggregation studies	Aggregation responses were preserved across plasticizer systems.	Few standardized datasets	Very low
Plasticizer migration	3–4 analytical chemistry studies	DEHP migration consistently exceeded DINCH and DEHT.	Limited matrix-specific datasets	Moderate
Modeled cumulative exposure	Deterministic modeling	Modeled exposure followed DEHP > DINCH > DEHT, with higher weight-normalized exposure in pediatric and neonatal scenarios.	Model-based estimates, not measured in vivo exposure	Low

Certainty was assessed using an adapted GRADE approach for mechanistic, analytical, and preclinical evidence. The main limitations were indirectness, small sample size, heterogeneity in platelet storage platforms and assay methods, and lack of direct clinical transfusion outcomes stratified by plasticizer class. Detailed domain-level judgments are provided in the Supplementary Material. DEHP, di(2-ethylhexyl) phthalate; DEHT, di(2-ethylhexyl) terephthalate; DINCH, diisononyl cyclohexane-1,2-dicarboxylate; PAC-1, activated GPIIb/IIIa binding antibody.

**Table 4:** Quantitative comparison of platelet metabolic and functional endpoints in DEHP versus non-DEHP storage systems.

Study	Platelet Product/System	Endpoint	Storage Day	DEHP	Non-DEHP	Interpretation
Snyder et al., 1992 [11]	Random-donor platelet concentrates	pH	Day 5	7.44 ± 0.13	7.31 ± 0.21	Comparable
Lagerberg et al., 2015 [9]	Pediatric platelet concentrates	pH	Day 7	6.93 ± 0.07	7.01 ± 0.04	Higher in non-DEHP
Lagerberg et al., 2015 [9]	Pediatric platelet concentrates	CD62P-positive platelets (%)	Day 7	22.3 ± 3.4	12.9 ± 1.5	Lower in non-DEHP
Lagerberg et al., 2015 [9]	Pediatric platelet concentrates	Annexin V-positive platelets (%)	Day 7	25.6 ± 2.4	16.3 ± 0.9	Lower in non-DEHP
Lagerberg et al., 2015 [9]	Pediatric platelet concentrates	Glucose (mmol/L)	Day 7	13.0 ± 1.5	14.4 ± 1.0	Higher in non-DEHP
Lagerberg et al., 2015 [9]	Pediatric platelet concentrates	Lactate (mmol/L)	Day 7	20.8 ± 3.0	18.3 ± 2.3	Comparable
Van Aelst et al., 2024 [16]	Buffy coat platelet concentrates in PAS-E	pH	Day 5	≈7.05 ± 0.05	≈7.08 ± 0.04	Comparable
Van Aelst et al., 2024 [16]	Buffy coat platelet concentrates in PAS-E	CD62P-positive platelets (%)	Day 5	≈32 ± 4	≈30 ± 3	Comparable
Van Aelst et al., 2024 [16]	Buffy coat platelet concentrates in PAS-E	Annexin V-positive platelets (%)	Day 5	≈6.5 ± 1.0	≈6.0 ± 0.8	Comparable
Van Aelst et al., 2024 [16]	Buffy coat platelet concentrates in PAS-E	Glucose (mmol/L)	Day 5	≈11.8 ± 0.9	≈12.1 ± 1.0	Comparable
Van Aelst et al., 2024 [16]	Buffy coat platelet concentrates in PAS-E	Lactate (mmol/L)	Day 5	≈16.5 ± 1.8	≈16.0 ± 1.6	Comparable

**Table 5:** Random-effects pooled estimates for platelet pH on day 7.

Endpoint	Timepoint	Number of Studies (k)	Model	Pooled Mean Difference (Non-DEHP–DEHP)	95% CI	τ^2^	I^2^ (%)	Q (df)	*p* (Heterogeneity)	Interpretation
Platelet pH (22 °C unless otherwise specified)	Day 7	2	Random-effects (DerSimonian–Laird)	+0.025 pH units	−0.081 to +0.131	0.0048	83.1	5.92 (df = 1)	0.015	No statistically significant difference in day 7 platelet pH between DEHP and non-DEHP systems; substantial between-study heterogeneity observed.

**Table 6:** Study-level quantitative comparison of platelet activation and aggregation following storage in DEHP-plasticized and DEHP-free systems.

Study (Reference)	Endpoint (Legend-Ready)	Assay/Method	Storage Day	DEHP (Mean ± SD; *n*)	Non-DEHP (Mean ± SD; *n*)	Mean Difference (Non-DEHP–DEHP)	95% CI (MD)	Effect Direction *	Notes
Snyder et al., 1992 [11]	Light transmission aggregation (Epinephrine + ADP)	Light transmission aggregometry	Day 5	68.4 ± 9.2 (*n* = 8)	66.7 ± 8.5 (*n* = 8)	−1.7	−10.5 to +7.1	≈ Equivalent	Direct functional aggregation assay
Larsson et al., 2021 [10]	PAC-1 binding (ADP-stimulated)	Flow cytometry	Day 7	26.6 ± 7.6 (*n* = 8)	26.9 ± 7.3 (*n* = 8)	+0.3	−7.0 to +7.6	≈ Equivalent	No impairment with non-DEHP
Larsson et al., 2021 [10]	PAC-1 binding (Collagen-stimulated)	Flow cytometry	Day 7	30.4 ± 7.5 (*n* = 8)	30.0 ± 7.6 (*n* = 8)	−0.4	−7.8 to +7.0	≈ Equivalent	Comparable activation response
Larsson et al., 2021 [10]	PAC-1 binding (Thrombin-stimulated)	Flow cytometry	Day 7	16.6 ± 5.5 (*n* = 8)	17.5 ± 5.4 (*n* = 8)	+0.9	−4.4 to +6.2	≈ Equivalent	Preserved signaling across systems

* Effect direction was determined based on the mean difference (non-DEHP–DEHP) and corresponding 95% confidence interval. “≈Equivalent” indicates that the confidence interval includes zero. Interpretations are descriptive and do not imply clinical superiority.

**Table 7:** Exploratory/descriptive study-level effect estimates for PAC-1 activation markers in DEHP versus non-DEHP platelet storage systems.

Endpoint	Timepoint/Storage Day	Studies (k)	Mean Difference (Non-DEHP–DEHP)	95% CI
PAC-1 (ADP)	Day 7	1 [10]	+0.3	−7.0 to 7.6
PAC-1 (Collagen)	Day 7	1 [10]	−0.4	−7.8 to 7.0
PAC-1 (Thrombin)	Day 7	1 [10]	+0.9	−4.4 to 6.2

Legend: Values represent descriptive single-study estimates derived from [10]. Mean differences are calculated as the non-DEHP minus the DEHP condition. Across all agonists (ADP, collagen, and thrombin), the confidence intervals include zero, indicating no statistically clear differences in agonist-induced PAC-1 binding between plasticizer systems. No meta-analysis was performed due to the availability of only a single study (abbreviations: PAC-1, activated glycoprotein IIb/IIIa binding antibody.

**Table 8:** Surface-area-normalized plasticizer migration into platelet concentrates across DEHP- and non-DEHP-storage systems.

Blood Component	Plasticizer	Storage Day	Migration Metric	Value	Units
Platelet concentrate (m-PC)	DEHP	Day 1	Plasticizer equivalent concentration	~1.80	µg/dm^2^/mL
Platelet concentrate (m-PC)	DINCH	Day 1	Plasticizer equivalent concentration	~0.30–0.40	µg/dm^2^/mL
Platelet concentrate (m-PC)	DEHT	Day 1	Plasticizer equivalent concentration	~0.15–0.25	µg/dm^2^/mL

Abbreviations: PC, platelet concentrate; Legend: data derived from Thelliez et al. [3]. Values represent surface-area-normalized plasticizer migration into platelet concentrates during early storage (dday 1). Approximate values (indicated by “~”) reflect reported ranges or averaged estimates. Compared with DEHP, DINCH and DEHT demonstrate substantially lower migration, with reductions of approximately 4–6-fold and ~8-fold, respectively. These gradients were consistent across labile blood products and reflected differences in the physicochemical properties and leachability of plasticizers. Migration differences are interpreted as mechanistic exposure differentials and provide the quantitative basis for downstream deterministic transfusion–exploratory exposure modeling.

**Table 9:** Exploratory modeling of cumulative plasticizer exposure from platelet transfusion.

Recipient	Transfusion Scenario	Plasticizer	Cumulative Exposure	Cumulative Dose	Interpretation
Adult	70 kg; 300 mL/day for 7 days	DEHP	22,680 µg	0.324 mg/kg	Highest exposure
Adult	70 kg; 300 mL/day for 7 days	DINCH	4410 µg	0.063 mg/kg	Lower exposure
Adult	70 kg; 300 mL/day for 7 days	DEHT	2520 µg	0.036 mg/kg	Lowest exposure
Pediatric	20 kg; 200 mL/day for 7 days	DEHP	15,120 µg	0.756 mg/kg	Highest exposure
Pediatric	20 kg; 200 mL/day for 7 days	DINCH	2940 µg	0.147 mg/kg	Lower exposure
Pediatric	20 kg; 200 mL/day for 7 days	DEHT	1680 µg	0.084 mg/kg	Lowest exposure
Neonate	3 kg; 30 mL/day for 7 days	DEHP	2268 µg	0.756 mg/kg	Highest exposure
Neonate	3 kg; 30 mL/day for 7 days	DINCH	441 µg	0.147 mg/kg	Lower exposure
Neonate	3 kg; 30 mL/day for 7 days	DEHT	252 µg	0.084 mg/kg	Lowest exposure

Legend: Modeled cumulative plasticizer exposure from repeated platelet transfusion scenarios derived from migration coefficients reported in Table 8 [3]. Scenario-level parameters, including body weight, transfused volume, duration, and container surface area, are constant within each population group and are therefore presented once per block. Exposure calculations incorporate migration rate, contact surface area, transfused volume, and recipient body weight. Results are expressed as daily and cumulative doses in milligrams per kilogram across adult, pediatric, and neonatal populations. The exposure model reflects deterministic projections based on in vitro migration data and does not represent the measured in vivo toxicologic dose.

## Data Availability

The data supporting this study are available within the article and Supplementary Materials. Additional data are available from the corresponding author upon reasonable request. All primary data sources were peer-reviewed publications listed in the references.

## Supplementary Materials

The following supporting information can be downloaded at https://www.mdpi.com/article/10.3390/cells15141276/s1; Table S1. Detailed methodological appraisal of the included studies, including study characteristics, sample size categories, risk-of-bias domains, assay validation, reporting completeness, and study-specific limitations supporting the adapted risk-of-bias and certainty-of-evidence assessments.

## Abbreviations

The following abbreviations are used in this manuscript:

ADP	Adenosine diphosphate
BTHC	Butyryl trihexyl citrate
CD62P	P-selectin platelet activation marker
CI	Confidence interval
DEHP	Di-2-ethylhexyl phthalate
DEHT	Di 2-ethylhexyl terephthalate
DINCH	Diisononyl cyclohexane 1,2 dicarboxylate
ECHA	European Chemicals Agency
EMA	European Medicines Agency
EU	European Union
FDA	U.S. Food and Drug Administration
HLA	Human leukocyte antigen
JC-1	Tetrachloro tetraethylbenzimidazolyl carbocyanine iodide dye
PAC-1	Activated GPIIb IIIa binding antibody
PAS	Platelet additive solution
PC	Platelet concentrate
PVC	Polyvinyl chloride
RBC	Red blood cell
REACH	Registration, Evaluation, Authorization, and Restriction of Chemicals
SCENIHR	Scientific Committee on Emerging and Newly Identified Health Risks
SD	Standard deviation
SSP	Storage solution for platelets, platelet additive solution E
SVHC	Substance of very great concern
TDI	Tolerable daily intake
TMH	Transfusion medicine and hemotherapy
TOTM	Tri 2 ethylhexyl trimellitate
WHO	World Health Organization
