# Supplementary material for: Platelet Storage Quality, Plasticizer Migration, and Transfusion Exposure Risk in DEHP Versus Non-DEHP Blood Storage Systems: A Mechanistic and Quantitative Comparative Analysis
Source: Cells. 2026 Jul 16;15(14):1276. doi: 10.3390/cells15141276 (PMC13406900; doi:10.3390/cells15141276)
Supplement: Supplementary file 1 [file cells-15-01276-s001.zip › cells-4328112-supplementary.pdf]

cells

MDPI

Supplementary Material

Supplementary Table S1.

SL No.	Study (Author, Year)	Study Type	Plasticizer System Evaluated	Endpoint Domain	Contribution to Review	PRISMA Status
1	Kerkhoffs et al., 2006	Randomized clinical study	PAS platelet storage	Clinical efficacy	Clinical context for platelet storage	Contextual evidence
2	Tobian et al., 2014	Clinical transfusion study	PAS apheresis platelets	Clinical outcomes	Clinical safety context	Contextual evidence
3	Tickner et al., 2001	Toxicologic review	DEHP	Toxicologic risk	Plasticizer safety context	Contextual evidence
4	Thelliez et al., 2023	Analytical migration study	DEHP vs DINCH vs DEHT	Migration	Exposure quantification	Quantitative synthesis
5	European Commission SCENIHR, 2015	Regulatory assessment	DEHP in medical devices	Regulatory risk	Regulatory framework	Contextual evidence
6	U.S. FDA, 2002	Regulatory report	DEHP medical devices	Exposure risk	Regulatory context	Contextual evidence
7	World Health Organization, 2012	Policy guideline	Blood safety	Clinical policy	Transfusion safety context	Contextual evidence
8	Rubin and Schiffer, 1976	Migration study	DEHP	Plasticizer migration	Exposure quantification	Contextual evidence
9	Buchta et al., 2003	Donor exposure study	DEHP	Plasticizer exposure	Toxicologic exposure	Contextual evidence
10	Koch et al., 2005	Toxicologic metabolism study	DEHP metabolites	Toxicologic metabolism	Exposure biology	Contextual evidence
11	Snyder et al., 1992	Experimental platelet storage	DEHP vs BTHC	pH, aggregation	Mechanistic comparison	Quantitative synthesis
12	Lagerberg et al., 2015	Platelet storage study	DEHP vs DINCH	Activation, apoptosis, metabolism	Mechanistic comparison	Quantitative synthesis
13	Larsson et al., 2021	Biomaterial storage evaluation	DEHP vs DEHT	Metabolism, migration	Plasticizer substitution	Quantitative synthesis
14	Lotens et al. 2026	Platelet storage study	PAS storage systems	Platelet function	Storage biology	Quantitative synthesis
15	Zimring et al., 2016	Metabolomics study	Platelet storage	Metabolic signatures	Mechanistic platelet biology	Contextual evidence
16	Rebulla, 2005	Clinical review	Platelet transfusion	Refractoriness	Clinical interpretation	Contextual evidence

 https://doi.org/10.3390/10.3390/cells15141276

17	Forest et al., 2016	Clinical transfusion review	Platelet refractoriness	Clinical management	Clinical interpretation	Contextual evidence
18	Cohn, 2020	Hematology review	Platelet refractoriness	Clinical mechanisms	Clinical interpretation	Contextual evidence
19	Couvidou et al., 2023	Immunology study	Anti-HLA antibodies	Platelet refractoriness	Immunologic mechanisms	Contextual evidence
20	Hagino et al., 2021	Clinical review	Non-immune refractoriness	Platelet clearance	Clinical mechanisms	Contextual evidence
21	van der Meer and de Korte, 2018	Review article	PAS systems	Platelet storage	Storage biology context	Contextual evidence
22	Bashir et al., 2014	Review article	PAS development	Storage strategies	Clinical context	Contextual evidence
23	EDQM Blood Guide, 2025	Regulatory guideline	Blood component preparation	Regulatory standards	Transfusion regulation	Contextual evidence
24	Basu et al., 2021	Platelet additive solution study	PAS vs plasma	Platelet increments	Clinical platelet storage	Contextual evidence
25	Stephenson et al., 2025	Biomaterial storage study	DEHT bags	RBC storage quality	Biomaterial substitution	Contextual evidence
26	Page et al., 2021	Reporting guideline	PRISMA 2020	Systematic review reporting	Methodological framework	Reporting guideline
27	Plaza et al., 2012	Platelet pooling study	Non-DEHP container	Platelet function	Storage system evaluation	Quantitative synthesis
28	Van Aelst et al., 2024	Platelet additive solution study	SSP+ vs T-PAS+	Platelet storage	Storage biology	Quantitative synthesis
29	Pötzl et al., 2024	Toxicologic plasticizer study	BPA analogs	Steroidogenesis	Plasticizer toxicity	Contextual evidence
30	European Chemicals Agency, 2008	Regulatory classification	DEHP SVHC	Toxicologic classification	Regulatory context	Contextual evidence
31	EU Medical Device Regulation, 2017	Regulatory legislation	Medical device plasticizers	Regulatory framework	Regulatory context	Contextual evidence
32	U.S. FDA, current webpage	Regulatory safety communication	DEHP medical devices	Exposure risk	Regulatory context	Contextual evidence
33	U.S. FDA Safety Assessment, 2001	Toxicologic assessment	DEHP medical devices	Exposure risk	Regulatory toxicology	Contextual evidence
34	European Medicines Agency, 2014	Regulatory guideline	Phthalate excipients	Toxicologic exposure	Regulatory framework	Contextual evidence

https://doi.org/10.3390/xxxxx

Supplementary Table S1. Detailed methodological appraisal of the included studies, including study characteristics, sample size categories, risk-of-bias domains, assay validation, reporting completeness, and study-specific limitations supporting the adapted risk-of-bias and certainty-of-evidence assessments.
